# Early or Delayed Onset of Food Intake in Time-Restricted Eating: Associations with Markers of Obesity in a Secondary Analysis of Two Pilot Studies

**DOI:** 10.3390/ijerph18189935

**Published:** 2021-09-21

**Authors:** Dorothea Kesztyüs, Petra Cermak, Tibor Kesztyüs, Anne Barzel

**Affiliations:** 1 Institute of General Practice, Ulm University Medical Center, Albert-Einstein-Allee 23, 89081 Ulm, Germany; petra-1.cermak@uni-ulm.de (P.C.); anne.barzel@uni-ulm.de (A.B.); 2 Department of Medical Informatics, Georg-August University, Von-Siebold-Straße 3, 37075 Göttingen, Germany; tibor.kesztyues@med.uni-goettingen.de

**Keywords:** time-restricted eating, circadian rhythm, anthropometry, health-related quality of life, adults, pilot study

## Abstract

Time-restricted eating (TRE) has rapidly gained interest in the public and the scientific community. One presumed mechanism of action is the adaptation of the eating–fasting rhythm to the evolutionary circadian rhythm of the metabolism. Study results regarding the suggestion that earlier beginning of food intake leads to better outcomes are heterogeneous. We conducted a secondary analysis of pooled data from two pilot studies on TRE to examine an association between the timing of onset of food intake with obesity-related outcomes. Participants (*n* = 99, 83 females aged 49.9 ± 10.8 years) were asked to restrict their daily eating to 8–9 h for three months. Tertiles of the onset of food intake were assessed for changes in anthropometry, blood lipid levels, and health-related quality of life. We detected no significant differences in outcomes between early (before 9:47), medium (9:47–10:50), and late onset (after 10:50) of food intake. However, the duration of the eating period was longest in the group with the earliest (8.6 ± 1.0 h) and shortest in the group with the latest onset (7.5 ± 0.8 h). Subsequently, fasting duration was longest in the last group (16.5 h). This may have compromised the results. More research is needed in this area to address this question.

## 1. Introduction

Despite numerous efforts and interventions, overweight and obesity are still on the rise, and according to the 2017–2018 National Health and Nutrition Examination Survey (NHANES), the adult obesity rate in the United States of America has passed the 40% margin [1]. According to the European Health Interview Survey (EHIS), the prevalence of obesity in adults in the European Union was 16% in 2020 [2], while the World Health Organization (WHO) reported 24% in 2016 [3]. Fasting is one out of several opportunities to lose weight but should not be mixed up with weight loss diets, e.g., very-low-calorie diets (VLCDs) with an energy intake of up to 600–800 kcal/day, as these are only applied temporarily to lose weight rapidly, while fasting follows a holistic approach [4]. Except for a weight reduction, these VLCDs result in few lifestyle or dietary changes and are often subject to weight cycling or the so-called YoYo effect [5]. In contrast to dieting, occasional fasting was no predictor of subsequent weight gain and onset of obesity in 692 female adolescents [6]. In recent years, intermittent fasting has received a lot of attention, with the focus set not exclusively on weight loss but also on health promotion and prevention and, above all, on healthy aging [7]. Time-restricted eating (TRE) as a special form of intermittent fasting aims to extend the overnight fasting time by shortening the daily period of food intake. The rationale behind the potential usefulness of TRE is the natural way humans ate tens of thousands of years before the invention of electric light and the 24 h availability of ready-to-eat highly palatable food [8]. Thus, TRE mimics the way of eating that is deeply rooted in our genes.

TRE offers a viable approach to gradual weight loss and the improvement of cardio-metabolic disease risk factors, which should be examined in more detail [9]. Most of the reliable information on limiting the period of food intake comes from animal studies, which show improvements in body weight, blood lipids, glucose, insulin and insulin sensitivity, and inflammation markers [10]. Results from human studies are still largely subject to limitations due to study design such as lack of control groups, short intervention and follow-up time, and small sample size [9]. Nevertheless, these studies provide valuable information that is necessary for the design of further studies on this topic. 

We conducted two pilot studies to examine the acceptance of a three-month 8–9 h TRE protocol in healthy workers at the Ulm University (2018) and secondly in patients with abdominal obesity in a general practitioners’ (GP) office (2019). Both studies revealed good feasibility and adherence to the eating/fasting protocol. Participants showed moderate weight loss and reductions in weight circumference and reported improved health-related quality of life (HRQoL) and sleep quality [11,12,13]. Emerging research focuses on the timing of TRE and suggests advantages of earlier onset of timeframes of food intake [14], and the different timing of the eating window seems to be an important factor with regard to the outcome [15]. Several physiological features suggest that an earlier start of food intake may be useful: food-induced thermogenesis is twice as high after meals in the morning as in the evening [16], the glucose tolerance of the body decreases continuously throughout the day after a high in the morning [17], and late-night eating leads to metabolic disturbances [18] and disruption of the circadian rhythm [17]. These physiological characteristics are reflected in research showing that later meal- and bedtimes are associated with a higher body fat percentage [19]. However, studies comparing TRE starting at different times of the day are scarce [20]. Hence, in the present article, we aim to examine the differences in obesity-related outcomes and HRQoL between tertiles of onset of food intake from pooled data of our pilot studies on TRE.

## 2. Materials and Methods

Both studies were planned in a pre-post design without controls with the main objective to test feasibility and adherence. The primary outcome was the percentage of days the fasting goal of at least 15 h was achieved according to the total number of days recorded by participants. Secondary outcomes were, among others, changes between pre- and post-measures in anthropometry, blood lipids, and health-related quality of life. Detailed information can be found elsewhere [11,13]. 

### 2.1. Recruiting

Participants of the first pilot study with employees of the Ulm University were recruited with flyers and an information lecture on behalf of occupational health management. Included were adult employees without known pre-existing metabolic conditions and without constraints of weight or waist circumference. Volunteers with diabetes type 1, hyperthyroidism, pregnancy, any eating disorder, or any condition where fasting was contraindicated were excluded from participation. The recruitment target was raised from 50 to 63 due to high interest but also in order to increase the number of male participants.

In the second pilot study, participants were recruited with flyers in the waiting room of a general practitioners’ office or invited by the physician during a consultation. Patients with abdominal obesity and any other component of the metabolic syndrome or diabetes type 2 not requiring insulin were included. The exclusion criteria in this study were the same as noted above. The target of 40 participants was reached within four weeks. 

### 2.2. Intervention 

Participants were instructed to reduce their daily period of food intake to 8–9 h, subsequently prolonging their overnight fast to 15–16 h. They were not asked to change their diet and were not restricted in the caloric content of their food. The intervention was implemented for a period of three months, from September to December 2018, for participants at the University and from February to May for patients in the GPs practice. During a baseline assessment, participants obtained detailed information about the intervention, aim, and scope and had the opportunity to clarify any arising questions or concerns. Additionally, they received a brochure with a comprehensive description of the eating–fasting procedure and generally understandable scientific background information. All participants were offered to contact the study team at any time in case of questions, problems, or discomfort.

### 2.3. Data Assessment

The pre- and post-measurements were conducted directly before the intervention started and at the end of the three months’ period. They comprised anthropometrics and questionnaires. For the period of the intervention, participants were given diaries to record data on eating and sleeping.

Anthropometric measurements were performed by trained staff according to a standardized protocol; details are described elsewhere [11,13]. Body mass index (BMI) was calculated as weight in kilogram divided by height in meter squared and overweight (≥25) and obesity (≥30) defined according to the specification of the World Health Organization. Waist-to-height ratio (WHtR), the division of waist circumference in centimeters by height in centimeters, was used to define abdominal obesity above a cut-off value of 0.5 as recommended [21].

During the study appointments, participants completed questionnaires to collect data on their lifestyle, health behaviour, and additionally, in the post version of the questionnaire, data regarding TRE, including side effects. Participants’ self-estimated daily duration of food intake before starting TRE was collected in the pre-questionnaire. Additionally, participants were asked which of the two chronotypes corresponded most to them: the lark, which goes to bed earlier and gets up earlier, or the owl, which goes to bed later and gets up correspondingly later. A third response option was neither. The health-related quality of life (HRQoL) was assessed pre and post with the visual analogue scale (VAS), taken from the EuroQol five-dimension (EQ-5D) questionnaire [22]. On this scale from 0 = the worst imaginable health status to 100 = the best imaginable health status, the respondent marks the status he or she currently perceives. Further detailed information on the questionnaires can be found in previous publications [11,13]. 

Each participant received a diary and was instructed to protocol the timing of the first and the last meal and the duration and quality of sleep of the previous night.

Finally, blood samples were drawn to determine blood lipids (low-density lipoprotein (LDL), high-density lipoprotein (HDL), total cholesterol, and triglycerides). All analyses were provided by certified contract laboratories.

### 2.4. Statistical Analysis 

Baseline descriptive values and follow-up data of each group are reported as means and standard deviations for continuous data. Differences between groups were tested depending on distribution and homogeneity of variance with one-way between-subjects ANOVA and Welch test and the Tukey HSD test for post hoc comparisons of significant differences or with the non-parametric Kruskal–Wallis test. 

Differences between males and females at baseline, follow-up, and with regard to the data from the diaries were examined applying *t*-test, Welch’s *t*-test, or Mann–Whitney U test according to distribution and heterogeneity in variance for continuous data and Fisher’s exact test for categorical data. 

Categorical data are reported as numbers and respective percentages and tested for overall differences with the chi-square test. Based on this, the respective *p*-values for differences between groups were obtained by transforming adjusted standardized residuals from the contingency table to chi-square values and applying a Bonferroni-corrected level of α = 0.0083 (2 × 3 contingency table). 

Differences between pre- and post-measures were tested with a general linear model for repeated measures and post hoc Tukey HSD test for normally distributed data or with the non-parametric Friedman test for pairwise comparisons and a Bonferroni correction for significant *p*-values.

Based on the data from the diaries, the mean value of the timing of the first meal, indicating the onset of the eating period, was calculated for each participant and subsequently, the entire group was divided into tertiles by the 33rd and the 66th percentile. Furthermore, the respective periods of fasting and food intake were calculated. The fasting periods were then used to calculate the percentage of days with achievement of the fasting goal of ≥15 h out of the total number of days recorded. Differences between tertiles were tested as described above for baseline and follow-up data.

The significance level, unless otherwise reported, was set at α = 0.05 for two-sided testing. All statistical analyses were performed with the statistical software packages IBM SPSS Statistics for Windows, Version 26.0. (IBM Corp., Armonk, NY, USA).

## 3. Results

Based upon two dropouts in each pilot study, data from 99 participants were included in this secondary analysis. Table 1 depicts the baseline characteristics of participants with regard to gender. 

Significant differences between male and female participants occurred in weight and waist circumference, HDL, triglycerides, and the LDL/HDL and triglycerides/HDL ratios derived from them.

The 33% percentile of onset of food intake was at 09:47 and the 66% percentile at 10:50. The earliest mean beginning was at 05:50 and the latest at 13:37. Each tertile consists of 33 participants. Baseline characteristics are depicted in Table 2.

At baseline, there were no statistically significant differences between tertiles except for the first tertile showing higher triglycerides and triglyceride/HDL ratios than the third tertile.

### 3.1. Follow-Up Data

From baseline to follow-up, some small changes occurred in the group of overweight participants, leading to significant differences between tertiles, but this significance was lost in pairwise comparisons. Differences in HRQoL were more pronounced than baseline leading to a significantly lower value in the third tertile as compared to the others. Triglycerides in the first tertile were marginally lowered so that the difference to the other tertiles was no longer significant; the same applies to the ratio of triglycerides/HDL. Detailed information is available in Table 3.

The differences between baseline and follow-up were mainly related to anthropometric measures such as weight, waist circumference, BMI, and WHtR. Weight decreased significantly in all tertiles, with no differences between groups. The same was true for waist circumference, BMI, and WHtR. HRQoL improved significantly in all tertiles from an increase of 5.8 ± 8.4 (*p* = 0.033) points in the first tertile to 10.1 ± 15.5 (*p* = 0.012) points in the third tertile, with differences between tertiles not reaching statistical significance. Total cholesterol was raised significantly in the second tertile from 5.3 ± 0.8 baseline to 5.6 ± 0.9 at follow up (*p* = 0.033).

Differences at follow-up between male and female participants corresponded to those at baseline in weight and waist circumference, as well as HDL, triglycerides, and the respective ratios derived from them. There were no significant differences in terms of changes reported as the respective Δ between baseline and follow-up data.

### 3.2. Diaries

Regarding the diaries of the participants, there were no significant differences between males and females. There were also no differences between the tertiles with respect to the number of documented days, sleep duration, and sleep quality. As expected, the timing of the last meal differed significantly between tertiles, but the third tertile showed the shortest eating and the longest fasting period and also the highest percentage of days with achieved fasting target. The exact information is given in Table 4.

**Figure 1:**
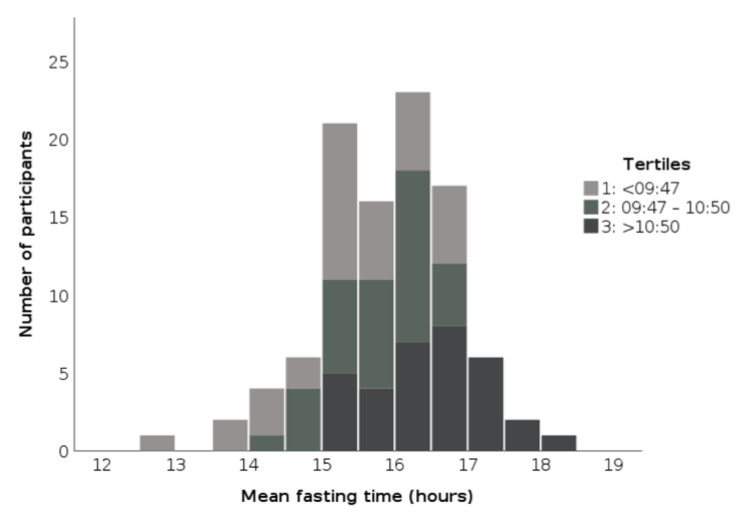
Mean period of fasting in the respective tertiles of the beginning of the eating phase of participants in the TRE pilot studies 2018/19.

**Figure 2:**
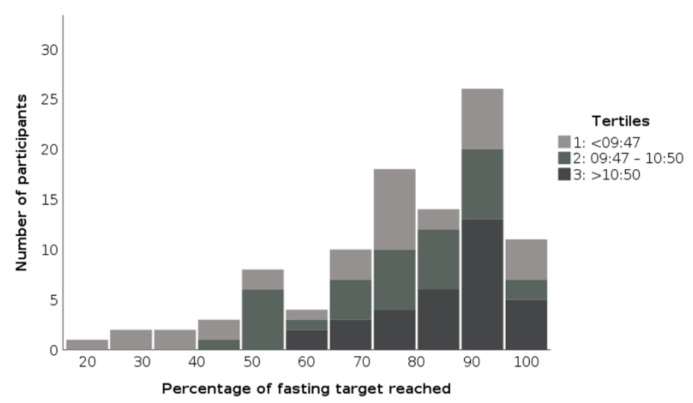
Illustration of the percentage of reaching the fasting target in relation to the tertiles of the beginning of the eating phase of participants in the TRE pilot studies 2018/19.

The differences in the length of the fasting phase and the percentage of achievement of the fasting target between the tertiles are visualized in Figure 1 and Figure 2.

## 4. Discussion

In this secondary data analysis of two pilot studies, we tried to figure out whether an earlier start of food intake in time-restricted eating (TRE) leads to better results with regard to anthropometric measures and health-related quality of life (HRQoL). The mean value of the onset of the eating phase was 08:49 in the first tertile, 10:16 in the second, and 12:03 in the third tertile. After three months of TRE, participants lost some weight (−1.5 ± 2.4 kg) and reduced waist circumference (−3.2 ± 3.6 cm) but without significant differences between the tertiles of onset of food intake. The same applies to the increase in HRQoL, although the values differed between +5.8 ± 8.4 points in the first tertile and +10.1 ± 15.5 points in the third tertile. Reasons for the absence of significance may be due to the relatively small sample size, which, divided into tertiles, was only 33 participants per tertile and the extent of variance. Other reasons may lie in the duration of fasting, which was significantly shorter in the first tertile than in the last one (15.4 vs. 16.5 h). The difference in mean time points of the first meal, as described above, amounted to 3.23 h between the first and third tertile, while the mean time of the last meal differed only by 2.12 h. Presumably, an earlier onset of the eating phase may have a similar positive effect on TRE outcomes as a shorter period of food intake. However, this assumption should be clarified in further studies, as the present study cannot provide evidence for this.

### 4.1. Results in the Context of Current Research

Modern lifestyle has substantially influenced eating habits, resulting in, among other consequences, a prolonged eating phase of more than 14 h daily [23]. The self-reported mean eating time before the start of the intervention in our sample was 12.4 ± 1.9 h, ranging from 9.0 to 18.0 h and without significant differences between tertiles. Regarding this from a circadian aspect, recognizing that eating and fasting entrain the peripheral clocks and hence impact metabolism with consequences for the development and progression of chronic disease [24,25], TRE offers a preventive and maybe even therapeutic approach. Despite a manageable number of TRE studies in humans, there is some evidence of the effects of daily fasting on body composition [24]. Our data analysis revealed an overall moderate weight loss of 1.5 ± 2.4 kg and a reduction in waist circumference of 3.2 ± 3.6 cm without significant differences between the early and late onset of eating. In a meta-analysis of TRE studies, 12 out of 19 studies reported changes in body weight with a mean weight loss of 0.90 ± 0.41 kg in 294 participants [26]. Unfortunately, waist circumference was not the subject of this meta-analysis, probably because it was monitored in only a few studies. A reduction of waist circumference would indicate reduced visceral fat, the metabolically most important fraction of body fat and crucial in the prevention of cardiovascular disease [27]. 

#### 4.1.1. Health-Related Quality of Life

A patient-reported outcome (PRO) is an instrument that measures treatment benefits or risks, particularly the status of a health condition reported by the patient directly [28]. Hence, changes in HRQoL represent the patient’s general perceptions of his physical, psychological, and social aspects of life in correlation with an intervention [28]. To date, there are few reports of HRQoL in TRE interventions, although HRQoL is, for example, an important concept used by the National Institute for Health and Care Excellence (NICE) to recommend treatments [29]. A study with 25 young men during Ramadan fasting failed to detect any changes in HRQoL compared to a control group [30]. Although Ramadan fasting is a type of time-restricted eating, the fasting period is from sunrise to sunset, the exact opposite of the period recommended in TRE interventions. In our study, HRQoL improved significantly by 7.8 ± 12.6 points and compared to Ramadan fasting, this may be partly due to eating–fasting in alignment with the circadian rhythm. Twelve weeks of TRE with an eight hours eating window improved health in subscales of the SF-3, such as emotional health and the health transition score, but not in general health in eleven participants compared to nine controls [31]. Using the SF-36 questionnaire, single aspects of the quality of life can be examined, while we decided to use the VAS, included in the EQ-5D questionnaire, which represents a general concept of HRQoL but with good discriminative ability [22]. A pilot study with ten sedentary older adults did not detect significant changes in HRQoL within four weeks of an eight hours feeding window [32], perhaps due to the small sample size and short intervention. A further study comprising 19 participants with type 2 diabetes in a 9 h TRE intervention for four weeks showed no changes in the AQoL-8D subscales [33], maybe due to the short intervention phase. Finally, three months of TRE with eight hours of eating resulted in improved values measured with the quality of life questionnaire of the World Health Organization (WHO-QoL) in 20 obese women as compared to control [34], confirming our results.

#### 4.1.2. Timing of Food Intake

There is increasing evidence that the timing of food intake influences the development of overweight, obesity, and metabolic disorders [35,36]. Hence, based on the evolutionary circadian rhythm of metabolism, early TRE is investigated on its presumed better effects on metabolic markers [14], and evidence is accumulating for the superiority of an earlier versus a later time window of food intake in TRE [15]. Currently, there are mainly smaller studies, often of a pilot character, that address this hypothesis, and against this background, most of the results presented below should therefore be viewed critically. Several studies of early TRE with stopping food intake before 19:00 h resulted in better weight loss, while late eating showed reduced beneficial effects [14]. A crossover study with 11 participants showed that eating three meals between 08:00 and 14:00 resulted in improved glucose response and detection of markers of circadian rhythmicity and autophagy as compared to a control schedule from 08:00 to 20:00 [37]. On the contrary, ad libitum TRE between 12:00 and 20:00 showed no significantly different outcomes in 116 participants compared to eating three structured meals per day, apart from a loss of fat-free mass in the TRE group, which the authors attributed to altered protein intake [38]. Considering the circadian rhythmicity of metabolism, the lack of effect of TRE, in this case, could be due to the late onset at 12:00, and the results of this trial should not be over-interpreted [39]. However, the timing of food intake may be as important as the calorie content of the meal consumed. An explanation for this hypothesis is given by the research of Richter et al. concerning differences in thermogenesis after high-calorie meals eaten at breakfast or dinner [16]. Effects on hunger, appetite for sweets, and resting energy expenditure during the day favor a high-calorie meal for breakfast rather than for dinner [16]. Furthermore, a more than twice as high diet-induced thermogenesis (DIT) after meals in the morning as in the evening, independent of the number of calories consumed, provides evidence for a diurnal course of DIT [16]. We assessed neither the amount of caloric intake nor meal size, which may have been helpful in detecting crucial differences between the early and late onset of eating in our TRE pilot studies. A randomized crossover study in 12 healthy adults revealed positive effects on weight, energy metabolism, and insulin of daytime (08:00–19:00) versus delayed (12:00–23:00) intake in the eight-week comparable meal schedule [35]. This difference of four hours in the onset of eating is 45 min more than what we found between early and late eaters in our sample. This may suggest that our groups were too close together to show differences in results plus the fact that we also experienced differences in the length of the eating phase between the first and last tertile. Another crossover study in which 22 participants were randomly assigned to a morning (08:00–09:30) versus an evening (20:00–21:30) isocaloric eating schedule showed potentially protective results against metabolic syndrome for eating in the morning [36], underscoring the importance of meal timing. Even without weight loss, early TRE (08:00–14:00) showed positive effects on glucose metabolism, blood pressure, oxidative stress, and appetite in the evening as compared to an extended eating pattern (08:00 to 18:00) in a crossover study with eight men with prediabetes [40]. Hence, results should not solely focus on changes in weight. An eight-hour TRE intervention with ad libitum eating between 10:00 and 18:00 led to decreased energy intake, weight loss, and reduced blood pressure in 23 adults with obesity, compared to a historical control [41]. Our participants were also allowed to eat ad libitum, which may positively influence the willingness to participate and adherence, but dietary advice could amplify the benefit for health. Finally, a systematic review and meta-analysis examined the effects of early TRE on the metabolic profile of adults with excess weight [42]. The authors concluded that although early TRE appears to have a beneficial effect on markers of the glucose–insulin metabolism, the results should be taken with caution because of the limited quality of the included studies [42]. 

#### 4.1.3. Sleep and Active Phase

To determine the individual phase of the circadian rhythm or the active phase, data on awakening and falling asleep are needed. Based on such data, Gill and Panda reported on the eating behavior of their participants and found that they tended to eat throughout the active phase, while the fasting duration was not much longer than the sleep duration [23]. In some studies of early TRE, participants were asked to maintain their usual sleep patterns [37,40]. Allison stated that “sleep–wake cycles were held constant” and reported mean values of sleep onset and offset, which differed slightly between daytime and delayed eating [25]. Regmi and Heilbronn point out that eating outside the phase of daily circadian rhythms causes metabolic desynchrony and may increase chronic disease risk, while TRE can concentrate total caloric intake to restricted periods during the active phase of the day [24]. Our participants’ eating window was predominantly during their active phase of the day; this is supported by the data on first and last meals. There is some evidence that circadian disruption due to light, activity, and food intake at night has adverse health effects [17]. TRE offers the opportunity to bring food intake back in line with the “natural” circadian rhythm. This includes, above all, the possibility to avoid eating in the (late) evening. Therefore, the timing of the onset of food intake seems to be important in TRE, especially to limit late eating, because the later the onset, the more the time window shifts to the late evening hours outside the natural active phase. 

Emerging themes in medicine are often introduced through pilot studies, and promising results need to be verified in high quality randomized controlled trials; this is true for our studies as well as for most of those discussed here.

### 4.2. Strength and Limitations

This study includes data from two pilot studies, one with initially 63 employees from Ulm University and another with 40 patients from a general practitioners’ office near Ulm. There were only two dropouts in each study; reasons were personal overload, illness, occupational stress, and, in one case, unknown. Overall, the willingness to participate and the adherence of the included participants were excellent, and only a few stated difficulties in implementing TRE. While anthropometric data were complete, the number of missing values in the questionnaires was extremely low, with one exception concerning the baseline data of HRQoL in the study at Ulm University, where the relevant question printed on the last backside of the questionnaire was overlooked by ten participants. Not all participants documented the full number of days in their diaries; however, the duration of the intervention was three months and the average number of days reported was 89 ± 7.

The main limitation was the lack of control group. This is partly attributable to the study design of a pilot study, but the implementation without external funding also resulted in a study plan tailored to the essentials. Another weakness is the lack of data on the time of the subjects’ falling asleep and waking up, which could have been used to determine the respective phase of the circadian rhythm at the onset of food intake. Instead, we asked participants about their self-assessment regarding an early or late chronotype (lark, owl, neither), but no statistically significant differences between tertiles were found here.

The results of our studies are subject to several types of bias. Firstly, due to the study design, randomization and blinding of staff and participants were not applicable, probably causing detection and performance bias. Attrition bias regarding missing outcome data was considerably low due to the small number of dropouts. However, selection bias occurred because participants actively decided to opt in, and the general practitioner invited eligible patients. Some items in the questionnaires and diaries may have been subject to bias due to social desirability. A recall bias can also not be excluded. Moreover, an obvious gender bias has to be considered due to an over-representation of women to men of 8:2.

## 5. Conclusions

This secondary data analysis did not reveal differences in outcomes between the earlier and delayed onset of eating in TRE, which may have possibly been modulated by the length of the eating phase. A later onset of about three hours with a shorter overall length of about one hour did not result in deviating reductions in weight and waist circumference or significant different improvements in HRQoL. Nevertheless, increases in HRQoL as a patient-reported outcome may emphasize the potential of TRE as a holistic measure to improve health, independent of the onset of the eating phase. 

It should be noted that in the absence of control group, the results of these two pilot studies only provide indications and need further validation in randomized controlled trials. Future research on TRE should focus on the significance of the onset of eating and the length of the eating phase, as these may be important keys to success.

## Figures and Tables

**Table 1:** Baseline characteristics of participants in the TRF pilot studies 2018/19.

	Female (*n* = 83)	Male (*n* = 16)	Total (*n* = 99)
Age, years m (sd)	49.5 (9.8)	49.1 (14.6)	49.9 (10.8)
University employees, n (%)	53 (63.9)	8 (50.0)	61 (61.6)
Weight, kg m (sd)	**76.2 (16.1) ^1^**	97.2 (22.0)	79.3 (18.5)
Waist circumference, cm m (sd)	**94.1 (14.2) ^2^**	107.3 (17.0)	95.7 (18.5)
BMI, kg/m^2^ m (sd)	27.8 (5.9)	30.0 (5.3)	28.0 (5.7)
WHtR, m (sd)	0.57 (0.09)	0.60 (0.09)	0.57 (0.09)
Overweight, *n* (%)	24 (28.9)	6 (37.5)	30 (30.3)
Obesity, *n* (%)	25 (30.1)	7 (43.8)	32 (32.2)
Abdominal obesity, *n* (%)	59 (71.1)	15 (93.8)	74 (74.7)
TCHOL, mmol/L m (sd)	5.5 (1.0)	5.0 (1.1)	5.4 (1.0)
HDL, mmol/L m (sd)	**1.7 (0.4) ^1^**	1.2 (0.4)	1.6 (0.4)
LDL, mmol/L m (sd)	3.5 (1.0)	3.3 (1.1)	3.5 (1.0)
Triglycerides, mmol/L m (sd)	**1.2 (0.6) ^1^**	2.0 (0.9)	1.4 (0.7)
LDL/HDL, m (sd)	**2.3 (0.9) ^3^**	2.9 (1.1)	2.3 (1.0)
Triglycerides/HDL, m (sd)	**0.8 (0.6) ^1^**	2.1 (1.5)	1.0 (0.9)
HRQoL, m (sd) *	71.8 (14.0)	72.5 (12.2)	72.2 (13.8)
Daily eating time, m (sd) **	12.4 (1.9)	12.8 (2.2)	12.4 (1.9)

*NOTE.* m—mean; sd—standard deviation; BMI—body mass index; WHtR—waist-to-height ratio; HRQoL—health-related quality of life; values in bold indicate significance; * 10 missing values; ** 1 missing value; ^1^ *p* < 0.001; ^2^ *p* = 0.002; ^3^ *p* = 0.013.

**Table 2:** Baseline characteristics of participants in the TRE pilot studies 2018/19 according to tertiles of breakfast.

	Tertiles of Beginning of the Eating Phase			
	Before 09:47	09:47–10:50	After 10:50	*p*-Value
Number of participants	33	33	33	
GPs practice, *n* (%)	15 (46)	7 (21)	16 (49)	**0.044 ^a^**
Age, years m (sd)	50.6 (11.7)	47.2 (10.3)	48.9 (10.5)	0.711
Female, *n* (%)	26 (85)	28 (89)	29 (88)	0.593
Weight, kg m (sd)	78.5 (20.4)	80.0 (19.4)	79.3 (16.1)	0.687
Waist circumference, cm m (sd)	96.0 (15.4)	94.4 (15.4)	96.7 (14.8)	0.594
BMI, kg/m^2^ m (sd)	27.9 (5.8)	27.5 (5.4)	28.7 (6.1)	0.563
WHtR, m (sd)	0.57 (0.08)	0.56 (0.09)	0.58 (0.10)	0.439
Overweight, *n* (%)	9 (27)	9 (27)	12 (36)	0.650
Obesity, *n* (%)	9 (27)	11 (33)	12 (36)	0.724
Abdominal obesity, *n* (%)	25 (76)	23 (70)	26 (79)	0.688
TCHOL, mmol/L m (sd)	5.6 (1.2)	5.3 (0.8)	5.4 (0.9)	0.413
HDL, mmol/L m (sd)	1.5 (0.4)	1.7 (0.5)	1.7 (0.4)	**0.049 ^a^**
LDL, mmol/L m (sd)	3.6 (1.2)	3.4 (0.8)	3.4 (0.9)	0.429
Triglycerides, mmol/L m (sd)	**1.7 (1.0) ^b^**	1.2 (0.5)	1.1 (0.4)	**0.020**
LDL/HDL, m (sd)	2.6 (0.9)	2.1 (0.8)	2.2 (1.1)	**0.040 ^a^**
Triglycerides/HDL, m (sd)	**1.5 (1.3) ^b^**	0.8 (0.4)	0.7 (0.4)	**0.014**
HRQoL, m (sd) *	74.6 (11.9)	75.5 (13.1)	66.6 (14.9)	**0.046 ^a^**
Daily eating time, m (sd) **	12.7 (1.9)	12.4 (1.8)	12.0 (2.0)	0.286
Chronotype, *n* (%) **				
Owl	6 (18)	11 (34)	11 (33)	
Lark	23 (70)	15 (47)	12 (36)	0.079
Neither	4 (12)	6 (19)	10 (30)	

*NOTE*. m—mean; sd—standard deviation; BMI—body mass index; WHtR—waist-to-height ratio; HRQoL—health-related quality of life; values in bold indicate significance or deviating groups, ^a^ significance lost in pairwise comparisons due to Bonferroni correction; ^b^ significant difference to the third tertile *p* = 0.020; * 10 missing values; ** 1 missing value.

**Table 3:** Follow-up results of participants in the TRE pilot studies 2018/19.

	**Tertiles of Beginning of the Eating Phase**			
	**Before 09:47**	**09:47–10:50**	**After 10:50**	***p*** **-Value**
Weight, kg m (sd)	77.2 (21.0)	78.4 (19.2)	77.7 (15.7)	0.698
Waist circumference, cm m (sd)	93.0 (14.9)	91.6 (14.7)	93.0 (13.7)	0.722
BMI, kg/m^2^ m (sd)	27.4 (5.9)	27.0 (5.3)	28.1 (5.9)	0.515
WHtR, m (sd)	0.56 (0.08)	0.54 (0.08)	0.56 (0.09)	0.460
Overweight, *n* (%)	5 (15)	7 (21)	14 (42)	**0.030 ^a^**
Obesity, *n* (%)	10 (30)	10 (30)	11 (33)	0.954
Abdominal obesity, *n* (%)	25 (76)	21 (64)	25 (76)	0.451
TCHOL, mmol/L m (sd)	5.7 (1.3)	5.6 (0.9)	5.6 (1.1)	0.864
HDL, mmol/L m (sd)	1.5 (0.4)	1.7 (0.5)	1.7 (0.4)	0.085
LDL, mmol/L m (sd)	3.7 (1.1)	3.4 (0.8)	3.6 (1.0)	0.579
Triglycerides, mmol/L m (sd)	1.6 (0.9)	1.5 (1.0)	1.2 (0.5)	0.293
LDL/HDL, m (sd)	2.6 (0.9)	2.2 (0.8)	2.3 (0.9)	0.099
Triglycerides/HDL, m (sd)	1.3 (1.1)	1.1. (1.3)	0.6 (0.4)	0.228
HRQoL, m (sd) *	79.9 (11.6)	82.8 (9.2)	**76.1 (11.9) ^c 1^**	**0.048**
Δ Weight, kg m (sd)	**−1.3 (2.8) ^b 2^**	**−1.6 (2.0) ^b 2^**	**−1.6 (2.5) ^b 3^**	0.895
Δ Waist circumference, cm m (sd)	**−3.0 (3.6) ^b 5^**	**−2.8 (3.7) ^b 4^**	**−3.7 (3.5) ^b 2^**	0.536
Δ BMI, m (sd)	**−0.5 (1.0) ^b 4^**	**−0.5 (0.7) ^b 4^**	**−0.6 (0.9) ^b 3^**	0.918
Δ WHtR, m (sd)	**−0.018 (0.022) ^b 5^**	**−0.017 (0.022) ^b 4^**	**−0.023 (0.022) ^b 5^**	0.523
Δ TCHOL, mmol/L m (sd)	0.12 (0.43)	**0.30 (0.48) ^b 6^**	0.23 (0.56)	0.320
Δ HDL, mmol/L m (sd)	0.03 (0.13)	0.00 (0.14)	−0.02 (0.17)	0.461
Δ LDL, mmol/L m (sd)	0.05 (0.43)	0.05 (0.38)	0.20 (0.59)	0.332
Δ Triglycerides, mmol/L m (sd)	−0.17 (0.44)	0.24 (0.80)	0.05 (0.41)	**0.044 ^a^**
Δ HRQoL, m (sd) **	**5.4 (8.4) ^b 6^**	**8.0 (13.1) ^b 7^**	**10.1 (15.5) ^b 8^**	0.479

*NOTE*. m—mean; sd—standard deviation; BMI—body mass index; WHtR—waist-to-height ratio; HRQoL—health-related quality of life; values in bold indicate significance or deviating groups, ^a^ significance lost in pairwise comparisons due to Bonferroni correction; ^b^ significant pre-post differences; ^c^ significant difference to the second tertile; ^1^ *p* = 0.044, ^2^ *p* = 0.001, ^3^ *p* = 0.007, ^4^ *p* = 0.003, ^5^ *p* < 0.001, ^6^ *p* = 0.011; ^7^ *p* = 0.005, ^8^ *p* = 0.004; * 2 missing values; ** 11 missing values.

**Table 4:** Diary of participants in the TRE pilot studies 2018/19.

	**Tertiles of Beginning of the Eating Phase**			
	**Before 09:47**	**09:47–10:50**	**After 10:50**	***p*** **-Value**
Number of documented days	87.7 (9.4)	88.9 (6.7)	89.4 (5.9)	0.409
Sleep duration, h m (sd)	7.4 (0.6)	7.5 (0.5)	7.3 (0.7)	0.206
Sleep quality, m (sd)	72.0 (15.1)	72.7 (12.9)	70.1 (15.6)	0.749
Time of first meal, m (sd)	**8.82 (0.91)**	**10.26 (0.33)**	**12.05 (0.69)**	**<0.001**
Time of last meal, m (sd)	**17.39 (1.33)**	**18.53 (0.76)**	**19.51 (0.84)**	**<0.001**
Eating phase, h m (sd)	8.6 (1.0)	8.3 (0.7)	**7.5 (0.8) ^a^**	**<0.001**
Fasting phase, h m (sd)	15.4 (1.0)	15.7 (0.6)	**16.5 (0.8) ^a^**	**<0.001**
Fasting target reached, % m (sd)	70.5 (23.7)	75.6 (16.0)	**85.5 (11.6) ^b^**	**0.013**

*NOTE*. h—hours; m—mean; sd—standard deviation; values in bold indicate significance or deviating groups; ^a^ significant difference to the first and second tertile *p* < 0.001; ^b^ significant difference to the first tertile *p* = 0.020.

## Data Availability

For data protection reasons (German Data Protection Act), datasets generated and analyzed during this study are only available on request from the responsible data manager, Tibor Kesztyüs.
